# Prognostic and Molecular Characterization of Metastatic Transverse Colon Cancer: Insights From a Single-Center Retrospective Study

**DOI:** 10.7759/cureus.75046

**Published:** 2024-12-03

**Authors:** Deniz Isik, Oguzcan Kinikoglu

**Affiliations:** 1 Medical Oncology, Kartal Dr. Lütfi Kirdar City Hospital, Health Science University, Istanbul, TUR

**Keywords:** left side colon carcinoma, metastatic transverse colon cancer, molecular mutation status, overall survival, right side colon carcinoma

## Abstract

Introduction: Metastatic transverse colon cancer (TCC) represents a unique subset of colorectal cancer with features of both right and left colon tumors due to its distinct embryologic origin. This study retrospectively analyzes the clinical, pathological, and molecular factors influencing survival outcomes in TCC patients treated at a single center.

Methods: For this study, we reviewed the files of 372 metastatic patients and analyzed the data of 71 patients with a diagnosis of TCC in detail. The remaining patients were patients with right or left colon tumors and we compared the overall survival (OS), molecular mutations (KRAS, NRAS, BRAF, MSI status), and clinicopathological features of our patients with transverse colon tumors with these patients.

Results: The median OS for TCC patients was 19.7 months, with metastasectomy and Eastern Cooperative Oncology Group (ECOG) performance status emerging as significant prognostic factors. Molecular analyses revealed KRAS mutations in 49% and BRAF mutations in 13% of TCC cases, aligning TCC closer to right-sided tumors in certain molecular characteristics. However, histopathologic diversity, including mucinous histology in 20% of TCC cases, indicated a need to consider TCC as a distinct entity.

Conclusion: These findings underscore the complex biological nature of TCC and the necessity for tailored therapeutic approaches, especially as survival rates remain suboptimal. Further multicenter, prospective studies are recommended to establish refined treatment strategies for TCC patients.

## Introduction

In colorectal tumors, the localization of the primary mass holds prognostic significance in both early and metastatic stages [1]. The terms “right colon” or “left colon” are used based on the origin site of the primary mass. Masses proximal to the splenic flexure are classified as the right colon, while those distal ones are considered the left colon. During embryonic development, right colon cancers (RCC) originate from the mid-gut, while left colon cancers (LCC) derive from the hind-gut, containing distinct anatomical, developmental, and carcinogenic differences [2]. Due to these fundamental differences, not only prognostic variations but also predictive differences in response to therapeutic agents, particularly in the metastatic stage, are observed between right and left colon tumors. In the metastatic stage, anti-EGFR (Epithelial Growth Factor Receptor) agents are preferred in RAS/BRAF wild-type tumors localized to the left colon, in addition to chemotherapy; meanwhile, in right colon tumors, chemotherapy is combined with Bevacizumab [3-6].

The transverse colon’s proximal two-thirds, including the hepatic flexure, originates from the mid-gut, while the distal one-third, containing the splenic flexure, is considered to derive from the hind-gut. Transverse colon cancer (TCC) comprises only 10% of all colon tumors [7]. Due to their heterogeneous embryological development, these tumors may exhibit behavior similar to either right or left colon tumors. Similar to right colon tumors, TCCs are often diagnosed at an advanced stage, such as T4, as bulky masses, due to the lack of specific symptoms until late stages [8]; moreover, microsatellite instability is frequently observed in TCC [9]. Conversely, like LCC, RAS/BRAF wild-type tumors in TCC have shown a favorable response to anti-EGFR therapy [10]. Past clinical studies mainly included these tumors as part of right colon tumors or excluded them altogether, leaving their response to treatment and prognosis relatively unknown [11,12]. A retrospective analysis revealed that tumors originating from the transverse colon exhibit different mutational profiles and consensus molecular subtype (CMS) frequencies compared to left- and right-sided colorectal cancers [9]. This study emphasized that transverse colon tumors display a distinct mutational character from right colon tumors, suggesting that they cannot be grouped with either side and should be considered as a unique entity.

For these reasons, our study aimed to investigate whether TCC differs from right or left colon tumors by assessing the clinical, pathological, and molecular prognostic factors known to be significant in colorectal cancer and examining whether anatomical localization shows any differences.

## Materials and methods

Patients

We evaluated a total of 372 patients diagnosed with metastatic colon cancer who were followed and treated in the Medical Oncology Department at Kartal City Hospital between 2005 and 2024. This study was designed retrospectively, cross-sectionally, and descriptively. Patient data were obtained from patient files containing detailed information in our oncology unit. We defined transverse colon tumors to include those from the hepatic flexure and extending distally to the splenic flexure. All patients were aged 18 or older and had metastatic disease. The localization of tumors and sites of metastasis were verified for all patients through both colonoscopy and radiological imaging methods. This study complied with the Declaration of Helsinki, and local ethics committee approval was obtained from Kartal City Hospital (Approval number: 2024/010.99/6/10, approval date: 26.07.2024).

We collected demographic data (sex, age, and Eastern Cooperative Oncology Group (ECOG) performance status at the time of primary diagnosis) and recorded whether surgery was performed on the primary tumor. We noted whether adjuvant therapy was administered in patients diagnosed at the local stage. Histopathological features (pT, pN, grade of differentiation, mucinous component, lymphovascular invasion (LVI)/perineural invasion (PNI), tumor localization, and metastatic sites) and molecular characteristics (KRAS, NRAS, BRAF, and microsatellite instability (MSI) status) were retrieved from the patients’ medical records. Additionally, any metastasectomies performed were also evaluated.

Statistical analysis

The primary outcome variable was overall survival (OS), the time from diagnosis to death from any cause. Chi-square and Fisher’s exact tests were used to compare categorical variables, such as age, gender, and ECOG performance status. The relationship between clinicopathologic parameters was initially assessed using univariate logistic regression. Univariate and multivariate analyses were performed using the Cox regression model to identify the best predictor variables. A p-value of < 0.05 was considered statistically significant for all tests. Statistical analyses were conducted using IBM SPSS Statistics 25.0 (IBM Corp., Armonk, New York, USA).

## Results

**Figure 1:**
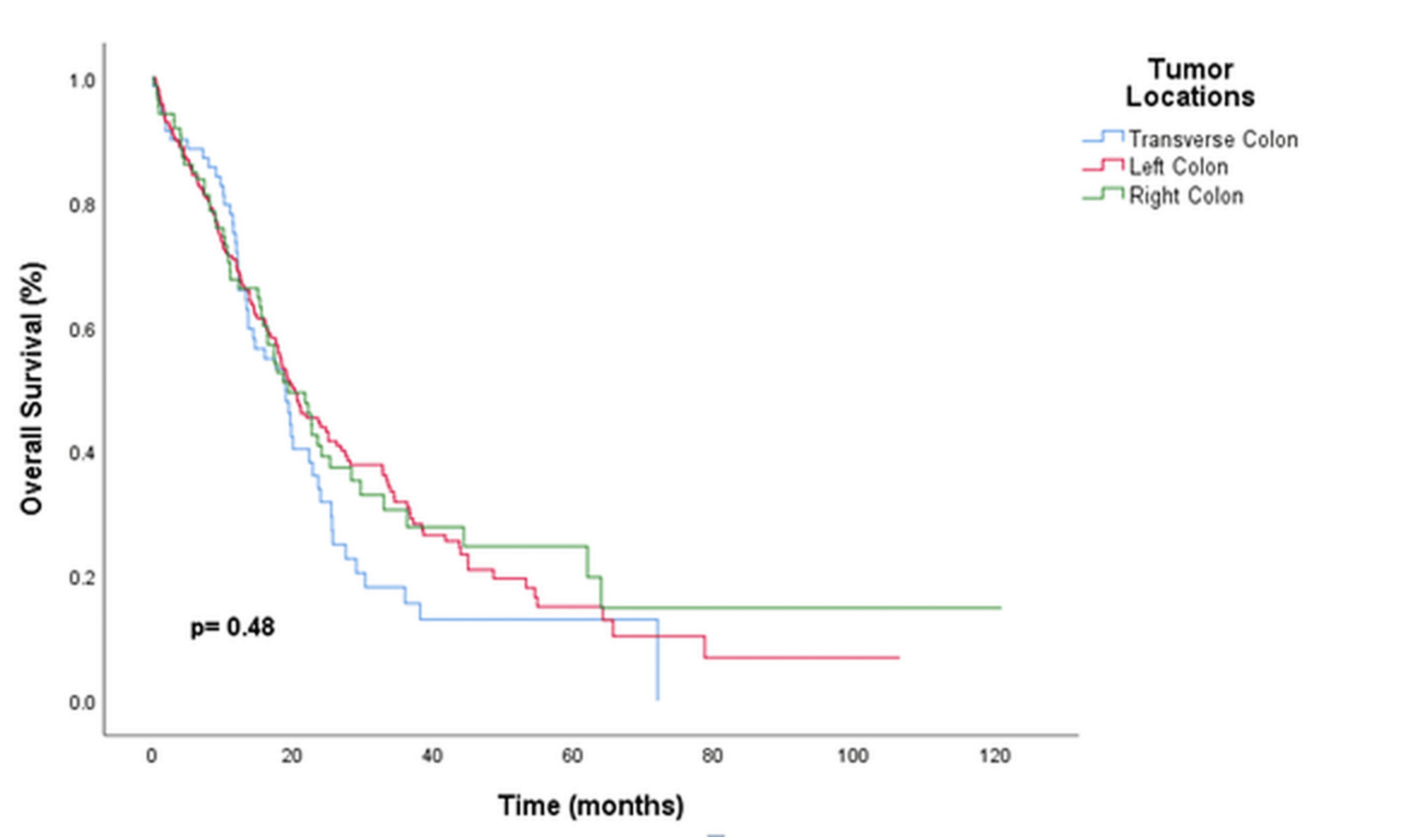
Kaplan-Meier Survival Curve by Tumor Location in Colon Cancer This Kaplan-Meier survival curve illustrates the overall survival rates of patients with colon cancer, segmented by tumor location. The lines represent survival outcomes for patients with tumors in the transverse colon (blue line), left colon (red line), and right colon (green line).

For this study, we reviewed the files of 372 metastatic patients and analyzed the data of 71 patients with a diagnosis of TCC in detail. The remaining patients were patients with right or left colon tumors and we compared the OS, molecular mutations (KRAS, NRAS, BRAF, MSI status), and clinicopathological features of our patients with transverse colon tumors with these patients. Among our patients, 88 had right colon tumors, 213 had left colon tumors, and the remaining 71 had metastatic transverse colon tumors, which were further analyzed in detail (Figure 1). The median age of the patients was 64 years (range: 37-85 years). Forty-four patients (61.9%) were male, and 27 (38.1%) were female. The clinicopathological characteristics of the patients are summarized in Table 1. Eighty percent of the patients had an ECOG score of 0, 1, or 2. At initial diagnosis, 74% of patients were in the metastatic stage, yet surgery on the primary lesion was performed in half of the cases. The most common histology observed was adenocarcinoma (78.9%), while mucinous adenocarcinoma pathology was present in 14 patients (19.7%).

**Table 1:** Demographic and Clinical Characteristics of Patients with Transverse Colon Cancer ECOG: Eastern Cooperative Oncology Group; n: number of patients; T stage: tumor stage; N stage: node stage

Patient Characteristics	Transverse Colon N=71 (%)
Sex (n=71)	
Male	44 (62)
Female	27 (38)
Age (median)	64
Range	37-85
ECOG (n=59)	
0	24 (41)
1	21 (35)
≥2	14 (24)
Histopathology (n=71)	
Adenocarcinoma	57 (80)
Mucinous adenocarcinoma	14 (20)
Initial Surgery (n=71)	
Yes	35 (49)
No	36 (51)
Initial stage ( n=71)	
1	0 (0)
2	5 (7)
3	13 (18)
4	53 (75)
Initial T stage (n=35)	
T1	2 (6)
T2	0 (0)
T3	18 (51)
T4	15 (43)
Initial N stage (n=35)	
N0	8 (23)
N1	14 (40)
N2	13 (37)
Adjuvant treatment (n=34)	
Yes	17 (50)
No	17 (50)

We performed KRAS testing in molecular analyses on 69 patients, finding 34 (49%) wild-type patients and 32 (46%) mutants. Of the 67 patients who underwent NRAS testing, 30 (45%) were wild-type, 34 (51%) were mutant, and three were reported as indeterminate. Similarly, BRAF mutation status was assessed in 36 patients, of whom 30 (84%) were wild-type, three were mutant, and three were indeterminate. Among the 27 patients for whom MSI status could be determined, 2 (7%) were MSI-high, three were indeterminate, and 22 were MSI-stable.

An analysis of metastatic sites revealed that liver metastasis was the most common, present in 78% of patients. The rate of lung metastasis was 31%, while 26% of patients had peritoneal involvement. Metastasectomy was performed in 18% of the patients. Patients who underwent metastasectomy were limited to those with no more than three liver metastases and four patients with more than two lung metastases. These selection criteria were determined by considering the surgical resectability of the metastases and the general performance status of the patients. Mutation analyses and metastatic sites of the patients are summarized in Table 2.

**Table 2:** Metastatic Spread, Genetic Profile, and Exclusion Criteria in Patients with Transverse Colon Cancer KRAS: Kirsten rat sarcoma viral oncogene homolog; NRAS: Neuroblastoma RAS viral oncogene homolog; BRAF: B-Raf proto-oncogene; MSI: microsatellite instability; n: number of patients

Metastasectomy (n=60)	
Yes	11 (18)
No	49 (82)
Liver metastasis (n=65)	
Yes	51 (78)
No	14 (22)
Lung metastasis (n=65)	
Yes	20 (31)
No	45 (69)
Peritoneal metastasis (n=65)	
Yes	17 (26)
No	48 (74)
KRAS status (n=69)	
Wild	34 (49)
Mutant	32 (46)
Unknown	3 (5)
NRAS status (n=67)	
Wild	30 (45)
Mutant	34 (51)
Unknown	3 (4)
BRAF status (n=36)	
Wild	30 (84)
Mutant	3 (8)
Unknown	3 (8)
MSI status ( n=27)	
MSI High	2 (7)
MSI Low	22 (82)
Unknown	3 (11)
Ex status ( n=71)	
Yes	52 (73)
No	19 (27)

During the follow-up period, 52 patients died. The OS of the patients was determined to be 19.7 months (95% CI: 14.8-24.6). OS among patients with transverse colon tumors was comparable to that of patients with right or left colon tumors; specifically, OS was 19.9 months for patients with right colon tumors and 20.1 months for those with left colon tumors (Figure 1). OS was significantly associated with ECOG performance status and the presence of metastasectomy. Patients with ECOG performance status of 0 or 1 had better survival compared to ECOG performance status of 2 and above (25 months vs 16.1 months; HR, 2.1; 95% CI, 1.06-4.1; p=0.004). Patients who underwent metastasectomy also had better survival in univariate analysis (38.1 months vs. 19.4 months; HR; 0.36; 95% CI: 0.14-0.87; p=0.02). No statistically significant differences were observed in OS based on initial diagnosis stage (local or metastatic), histology type (adenocarcinoma or mucinous adenocarcinoma), primary colon surgery, KRAS, NRAS, and BRAF status, or metastatic sites (p > 0.05) (Tables 3-4). The multivariate analysis confirmed that ECOG performance status (≥2 vs. 0-1; HR: 1.43; 95% CI: 1.07-1.92; p=0.014) and metastasectomy (yes vs. no; HR: 0.1; 95% CI: 0.01-0.83; p=0.03) remained independently associated with OS.

**Table 3:** Univariate Analysis of Factors Associated with Metastatic Transverse Colon Cancer Outcomes ECOG: Eastern Cooperative Oncology Group; n: number of patients; OR: odds ratio; CI: confidence interval; KRAS: Kirsten rat sarcoma viral oncogene homolog; NRAS: neuroblastoma RAS viral oncogene homolog; BRAF: B-Raf proto-oncogene

Patient characteristics	Category	n	(%)	HR (95% CI)	P
ECOG	0 and 1	45	(76)	Reference	NA
	≥2	14	(24)	12.1 (1.06-4.1)	0.04
Initial Surgery	Yes	35	(49)	Reference	NA
	No	36	(51)	0.99 (0.59-1.64)	0.970
Histology	Adenocarcinoma	57	(80)	Reference	NA
	Mucinous adenocarcinoma	14	(20)	1.75 (0.60-5.14)	0.307
Stage at Diagnosis	Locally	18	(25)	Reference	NA
	Metastatic	53	(75)	1.25 (0.41-3.81)	0.694
KRAS	Wild	34	(49)	Reference	NA
	Mutant	32	(46)	0.84 (0.56-1.31)	0.446
NRAS	Wild	30	(45)	Reference	NA
	Mutant	34	(51)	0.88 (0.57-1.37)	0.575
BRAF	Wild	30	(84)	Reference	NA
	Mutant	3	(8)	1.21 (0.80-1.82)	0.352
Lung Metastasis at Diagnosis	No	45	(69)	Reference	NA
	Yes	20	(31)	0.69 (0.40-1.20)	0.198
Liver Metastasis at Diagnosis	No	14	(22)	Reference	NA
	Yes	51	(78)	1.00 (0.60-1.67)	0.990
Peritoneal Metastasis at Diagnosis	No	48	(69)	Reference	NA
	Yes	17	(31)	0.78 (0.42-1.42)	0.420
Metastasectomy	No	49	(82)	Reference	NA
	Yes	11	(18)	0.36 (0.14-0.87)	0.02

**Table 4:** Multivariate Analysis of Factors Influencing Outcomes in Metastatic Transverse Colon Cancer ECOG: Eastern Cooperative Oncology Group; n: number of patients; OR: odds ratio; CI: confidence interval; KRAS: Kirsten rat sarcoma viral oncogene homolog; NRAS: neuroblastoma RAS viral oncogene homolog; BRAF: B-Raf proto-oncogene

Patient characteristics	Category	n	(%)	HR (95% CI)	P
ECOG	0 and 1	45	(76)	Reference	NA
	≥2	14	(24)	13.4(1.7-6.6)	<0.01
Initial Surgery	Yes	35	(49)	Reference	NA
	No	36	(51)	0.99(0.59-1.65)	0.971
Histology	Adenocarcinoma	57	(80)	Reference	NA
	Mucinous adenocarcinoma	14	(20)	1.76(0.81-4.94)	0.324
Stage at Diagnosis	Locally	18	(25)	Reference	NA
	Metastatic	53	(75)	1.29(0.51-3.97)	0.714
KRAS	Wild	34	(49)	Reference	NA
	Mutant	32	(46)	0.79(0.52-1.22)	0.305
NRAS	Wild	30	(45)	Reference	NA
	Mutant	34	(51)	0.90(0.57-1.43)	0.675
BRAF	Wild	30	(84)	Reference	NA
	Mutant	3	(8)	1.27(0.82-1.95)	0.269
Lung Metastasis at Diagnose	No	45	(69)	Reference	NA
	Yes	20	(31)	0.66(0.38-1.15)	0.143
Liver Metastasis at Diagnose	No	14	(22)	Reference	NA
	Yes	51	(78)	0.98(0.58-1.63)	0.943
Peritoneal Metastasis at Diagnose	No	48	(69)	Reference	NA
	Yes	17	(31)	0.83(0.44-1.57)	0.582
Metastasectomy	No	49	(82)	Reference	NA
	Yes	11	(18)	0.32(0.14-0.77)	0.01

## Discussion

Currently, the localization of the primary tumor is of significant importance in metastatic colon cancer, as the tumor’s location in either the right or left colon influences disease management and treatment options. However, considering the embryonic development, blood supply, and lymphatic drainage of the colon, simply classifying it as right or left alone is inadequate to fully capture the disease characteristics, indicating a need to examine more detailed subtypes of the colon. Research in this area continues to progress [7,9,13].

Our study assessed the impact of clinicopathological and molecular data on survival in stage 4 TCC patients. Although our study was retrospective, with limited sample size and certain challenges in accessing comprehensive patient data, our patient sample was consistent with the literature [8,9,11,14]. Transverse colon tumors have often been analyzed within the category of right colon tumors; however, they can display behaviors similar to both right and left colon cancers [15-17]. Consequently, it is challenging to provide a definitive approach to their treatment or make firm prognostic conclusions.

We examined the transverse colon as a distinct group, including 71 patients in our study. Most of our patients were male (61.9%), with an average age of 64. At diagnosis, 74% were in stage 4. In our center, the age at diagnosis, gender, and metastatic stage rates were similar among patients with right and left metastatic colon tumors. Although clear data on transverse colon tumors are limited in the literature, studies indicate that left colon tumors are more common in males compared to right colon tumors [18,19]. In our study, 14 patients had mucinous histology. We observed a similar rate (21%) of mucinous histology among right colon tumor patients but a markedly lower rate (6%) in left colon tumors. Large studies report that the rate of mucinous histology is approximately 19% in right colon tumors and 4% across all colon tumors [20]. Conflicting reports exist regarding the prognosis of tumors with mucinous histology. Some studies have reported an adverse impact on survival, while others suggest no significant effect [21,22]. In our patients with mucinous histology, OS was similar to that of the broader patient group. The small patient sample may contribute to the lack of observed survival differences. Tumors originating in the right colon are known to exhibit MSI-H status in up to 30% of cases, whereas only about 2% of left colon tumors show MSI-H [23]. Due to the long study period and inclusion of older cases, we could not assess MSI status in many patients; however, the rate of MSI-H patients in our sample was 7%, similar to the left colon tumor rate (2%). Our molecular analysis revealed that 49% of patients had KRAS mutations, a rate comparable to left colon tumors [9,14]. BRAF mutations were present in 13% of patients, which aligns with findings from studies on right colon tumors [9].

We determined the OS in our study to be 19.7 months. This relatively low OS may be attributed to a high rate of patients with peritoneal metastasis, limited access to optimal modern therapies among patients diagnosed in earlier years, a high frequency of KRAS and BRAF mutations, and a limited number of metastasectomies. The treatment of metastatic colon cancer has improved considerably today, especially with the addition of anti-vascular endothelial growth factor (VEGF) and anti-epidermal growth factor (EGFR) antibodies to effective combination chemotherapy as a result of widespread molecular analysis. Recently, with the inclusion of immunotherapies in the treatment, a significant difference in progression-free survival has been achieved, especially in deficient DNA mismatch repair (dMMR) cases, compared to chemotherapy combinations.

Two studies similar to ours in the literature are noteworthy. A study by Küçükarda et al. [24] examined prognostic factors in operated TCC patients. Stage 4 patients were not included in this study. It was reported that transverse colon tumors showed a progression pattern more similar to right colon tumors in terms of molecular profile and prognosis, with BRAF mutation identified as a poor prognostic factor even in the early stages. This study aimed to investigate the effect of BRAF mutation on prognosis, especially in the non-metastatic stage, and patients in the metastatic stage were not included in the study, which is the opposite of our study. Therefore, it cannot present clinical, molecular, and OS data of metastatic transverse colon tumors as our study does, but it contains valuable information for the local stage. Another study by Roberto et al. [12] presented data from 97 stage 1-4 TCC patients. As in our study, most patients were male (61%), and 68% had an ECOG score of 0. This study reported an MSI-H rate of 26%, a KRAS mutation rate of 37%, and a BRAF mutation rate of 24%. High-grade and BRAF mutations were identified as factors adversely affecting OS. In this study, unlike our own study, all patients with transverse colon tumors, regardless of stage, were included in the study. It is a very valuable study in terms of mutational analysis and factors affecting OS. However, as we mentioned above, since our study only included metastatic patients, we present different information from other studies, especially with the data we present only at this stage.

There were some limitations to our study. First, this was a retrospective study conducted at a single oncology center. Second, we could not analyze all patients’ molecular data due to missing information in patient records. Some patients had missing molecular data, which introduced limitations in certain analyses of the study. The absence of molecular data may have affected the results, particularly in identifying prognostic factors and evaluating treatment approaches. Future research should focus more on completing molecular data and improving the management of missing data. Further large-scale, multicenter prospective studies are needed to validate the prognostic factors for TCC.

## Conclusions

The present study provides valuable insights into the clinicopathological characteristics of TCC patients. Our findings suggest that TCC possesses traits similar to both right and left colon tumors while also displaying unique characteristics, indicating the need for tailored treatment approaches for these tumors. However, we found OS to be lower due to the lack of detailed molecular analyses for older cases and the lower efficacy of historical treatments compared to current standards. Future studies should focus on large-scale, multicenter prospective trials involving a high number of patients with detailed molecular analyses. Specifically, examining the role of key molecular mutations, such as KRAS, NRAS, and BRAF, in TCC will provide deeper insights into the disease’s behavior and potential targeted therapeutic strategies. Such studies would help clarify prognostic factors and refine treatment approaches for TCC.
